# Genome sequences of the honey bee pathogens *Paenibacillus larvae* and *Ascosphaera apis*

**DOI:** 10.1111/j.1365-2583.2006.00694.x

**Published:** 2006-10

**Authors:** X Qin, J D Evans, K A Aronstein, K D Murray, G M Weinstock

**Affiliations:** * Human Genome Sequencing Center, Baylor College of Medicine Houston, TX, USA; † Bee Research Laboratory, USDA-ARS, BARC-E Beltsville, MD, USA; ‡ Honey Bee Unit, USDA-KSARC Weslaco, TX, USA

**Keywords:** host–pathogen interaction, American foulbrood, chalkbrood

## Abstract

Genome sequences offer a broad view of host–pathogen interactions at the systems biology level. With the completion of the sequence of the honey bee, interest in the relevant pathogens is heightened. Here we report the genome sequences of two of the major pathogens of honey bees, the bacterium *Paenibacillus larvae* (causative agent for American foulbrood disease) and the fungus *Ascosphaera apis*. (causative agent for chalkbrood disease). Ongoing efforts to characterize the genomes of these species can be used to understand and mitigate the effects of two important pathogens, and will provide a contrast with pathogenic, benign and freeliving relatives.

## Introduction

The completion of the genome sequence of the honey bee *Apis mellifera* (Honey Bee Genome Sequencing Consortium, 2006) represents a significant contribution toward understanding the social processes in this organism. A full understanding of the genetic implications and mechanisms of sociality requires a complementary description and understanding of the environment in which the organism exists. Infectious diseases are a component of such an environment that must be critically controlled in the dense population structures of a social organism. This is no exception with the honey bee and important pathogens have been described that can be devastating to a colony. Two of the most important are the bacterium *Paenibacillus larvae* and the fungus *Ascosphaera apis*. To appreciate the infectious processes of these microbes, and thus the resistance mechanisms developed by the honey bee (Evans *et al*., 2006), requires a complete elucidation of virulence factors and accessory elements needed for infection. To this end, we report draft genome sequences for these two pathogens as a prelude to more extensive genome annotation and functional analyses.

The spore-forming bacterium *Paenibacillus larvae* (Genersch *et al*., 2006) is the agent behind American foulbrood (AFB), a widespread larval pathogen of the honey bee, *Apis mellifera*. Young larvae (from the first and second instars) are highly susceptible to this disease, with the ingestion of as few as 10 infectious spores from virulent strains being sufficient to cause mortality (Brodsgaard *et al*., 1998). Infected bees generally die from the disease late in larval development, forming a dry ‘scale’ with approximately 2 × 10^9^ bacterial spores/bee (Shimanuki & Knox, 1997). Despite the observed virulence of *P. larvae* toward bees, many strains of this bacterium are relatively benign. These strains, once classified as separate subspecies (Heyndrickx *et al*., 1996), do not reflect distinct genetic lineages and there are great incentives from the standpoint of both diagnostic tests and research questions to identify those traits directly linked to virulence. A congener of *P. larvae*, *P. alvei*, is also found in bees but is considered to be a relatively benign species. The genus *Paenibacillus* as a whole is comprised mainly of soil-dwelling opportunistic bacteria. Among the *Bacillales*, *Paenibacillus* appears to be most closely related to the genus *Brevibacillus*. Bees mitigate this disease both through hygienic behaviour by adult workers and through larval resistance traits.

*Ascosphaera apis* (Qin *et al*., 1993; Anderson *et al*., 1998) is the fungal cause of the honey bee larval disease chalkbrood (Williams, 2000; Hornitzky, 2001). Spores of this fungus germinate within the digestive tract of bees, then begin fungal filamentous (mycelial) growth during the last instar of larval development. Dead larval and pupal bees appear chalky due to growth throughout the bee of mycelia. These chalky ‘mummies’ are highly infectious, and spores of this fungus often reinfect colonies via stored food supplies or direct transport to younger larvae by adult bees working within the nest. Adult bees reduce the effects of this fungus on the colony by frequently identifying and removing diseased individuals. The disease is associated with high brood density (productivity) and cooler outside temperatures.

## Results

Draft sequences of *P. larvae* and *A. apis* were produced by standard procedures and have been deposited at DDBJ/EMBL/GenBank under the project accession AARF00000000 (*P. larvae*) and AARE00000000 (*A. apis*). The versions described in this paper are the first versions, AARF01000000 and AARE01000000, respectively. Whole genome shotgun libraries were constructed in plasmid vectors and paired-end sequences were generated by the Sanger dideoxy-terminator technique and assembled using the Atlas and Phrap assembly programs as described (McLeod *et al*., 2004). *Paenibacillus larvae* posed a number of challenges, such as obtaining a pure strain and sequencing difficulties, as manifested by a relatively low rate of successful sequence reads (Table 1). Moreover, after assembling sequences containing 43 Mb of high quality bases, expected to represent about 10× coverage of the genome, many regions were only covered at 6× or lower coverage (as judged by the number of overlapping reads), suggesting selection against cloning these sequences in *Escherichia coli*. In any case, this assembly produced contigs that spanned over 4 Mb, the expected size for this genome, with an N50 length of 11 kb, which is sufficient contiguity to allow prediction of genes and operons. These contigs were linked into scaffolds by read-pairs that doubled this N50 length. Thus the genome is 4.0 Mb and is likely nearly completely represented in the assembly.

**Table 1:** *Paenibacillus larvae* genome assembly v2005–07–06

Reads	
Attempted reads	79 826
Passed reads	54 073 (68%)
Average trimmed read length	701
Passed Q20 bases	43.0 Mb (10.8× for 4.0 Mb genome)
Read overlap peak	9–12 (coverage 5–6×)
Assembly contigs	
Number of contigs	646
Total contig length	4 016 553 bp
Number of contigs = 2 kb	471
Total length of contigs = 2 kb	3 758 779 bp
Average contig length	6217 bp
N50 contig size	11 019 bp
Largest contig size	58 621 bp
Total assembled reads	47 144
Total assembled Q20 bases	33.0 Mb (8.3× for 4.0 Mb genome)
Scaffolds	
Number of scaffolds = 1 kb	349
Number of singleton scaffolds	197
Total size of singleton scaffolds	982 435 bp
Number of multiple-contig scaffolds	152
Number of contigs in multiple-contig scaffolds	436
Total size of multiple-contig scaffolds	3 033 342 bp
Total size of scaffold (including = 1 kb singletons)	4 015 777 bp
Average scaffold size	11 506 bp
N50 scaffold size	23 797 bp
Biggest scaffold	137 418 bp

This draft sequence was used for gene predictions by two programs, GeneMark (Besemer & Borodovsky, 2005) and GLIMMER2 (Delcher *et al*., 1999). With GeneMark the computation was performed with four different *Bacillus* genomes as training sets (*B. anthracis*, producing 4917 predicted genes; *B. cereus*, 4915; *B. halodurans*, 5006; *B. subtilis*, 5036) yielding about 5000 predicted genes for each. When GLIMMER2 was used (with self-training) 8503 genes were predicted, but almost all of the excess were very small open reading frames, less than 400 bp. This could be the result of frameshift errors in the draft sequence that may have less effect on GeneMark. The predicted genes were compared to sequences in GenBank with Blast and the resulting automated annotation is available at http://www.hgsc.bcm.tmc.edu/projects/microbial.

The draft sequence of *A. apis* was produced in much the same way (Table 2). In this case a higher rate of successful reads was obtained but there also appeared to be some selection against sequences. A total of over 156 Mb of high quality bases was produced and assembled into contigs that totaled 21.6 Mb in length. This represents 7× coverage but again, a lower value (4×) was obtained by read overlap analysis, indicating possible selection against reads in *E. coli*. The N50 length for these contigs was low, 3 kb, which can result from repeated sequences and/or low coverage. However these short contigs were joined into very long scaffolds with an N50 of 44 kb. Thus while the contigs are small, the scaffolds are suitable for gene predictions (in progress).

**Table 2:** *Ascosphaera apis* genome assembly v2005–07–06

Reads	
Attempted reads	256 859
Passed reads	204 025 (79.4%)
Average trimmed read length	675 bp
Total passed Q20 bases	156.2 Mb (7.3× for 21.5 Mb genome)
Read overlap peak	7 (coverage 4×)
Assembly contigs	
Number of contigs	8092
Total contig length	21 566 106 bp
Number of contigs = 2 kb	4619
Total = 2 kb contig length	16 148 594 bp
Average contig length	2665 bp
N50 size	2967 bp
Largest contig size	16 610 bp
Total assembled reads	168 066
Total assembled Q20 bases	113.4 Mb (5.3× for 21.5 Mb genome)
Average base coverage from ace	7.0×
Scaffolds	
Number of scaffolds = 1 kb	1627
Number of singleton scaffolds	880
Total size of singleton scaffolds	1 548 023 bp
Number of multiple-contig scaffolds	747
Number of contigs in multiple-contig scaffolds	7172
Total size of multiple-contig scaffolds	19 729 929 bp
Total size of scaffold (including = 1 kb singletons)	21 277 952 bp
Average scaffold size	13 078 bp
N50 scaffold size	44 063 bp
Biggest scaffold	195 321 bp

## Discussion

The genomic view of host–pathogen interactions moves these molecular studies from piecemeal strategies to the systems biology level. With the completion of the sequence of the honey bee, interest in these pathogens is intensified. Knowledge of the genetic makeup of two of the major pathogens of honey bees now allows a full program to be developed.

Analysis of these two sequences will follow familiar pathways. Annotation will include community involvement, using gene lists produced by automated procedures. For *P. larvae*, prokaryotic gene prediction programs have produced such gene lists already. For *A. apis* producing comprehensive gene lists is more of a challenge. Because of the diversity of fungal coding sequences, available genomes used to train prediction programs may not be representative. However, more fungal genomes are being sequenced, for example over 25 fungal genomes as part of the Fungal Genome Initiative (http://www.broad.mit.edu/annotation/fgi/). Thus there is likely to be more effective gene prediction options available in the future.

This program will focus on more fully annotating these two pathogen genomes, contrasting their genomic makeup with that of related microbes, and generating functional data related to their survival and pathogenicity in bees. Given the long-term relationship between these pathogens and honey bees, extensive data on pathology, and an opportunity to look at disease transmission and efficacy in a social setting, this system offers excellent opportunities for studying host–pathogen relationships. Moreover, the honey bee has a simpler immune defence system than other insects (Evans *et al*., 2006; Honey Bee Genome Sequencing Consortium, 2006), and thus offers an opportunity for analysis of a less complex host–pathogen interaction.

## Experimental procedures

### Source of DNA

The sequenced strain of *P. larvae* was isolated from scales collected from a single severely diseased colony from Berkeley, CA, USA (isolate BRL-230010). A suspension containing 1 × 10^8^ was heat shocked then used to inoculate replicate plates containing Brain-Heart Infusion agar (Difco, Franklin Lakes, NJ), supplemented with 0.1 mg/ml thiamine hydrochloride. Single colonies from this and two serial replatings were then grown at quantity on the same medium for genomic DNA.

DNA was isolated from scraped cells using an overnight incubation in a final concentration of 100 µg/ml proteinase K, followed by NaCl/CTAB precipitation and phenol/chloroform washes. DNA was precipitated using isopropanol, pulled from solution with a glass rod, and washed gently with EtOH before drying and suspension at approximately 100 ng/µl concentration. Extractions were also carried *in situ* in agarose gels using pronase. Slices from gel-extracted DNA were equilibrated in TBE buffer for PFGE analyses of genome size (below), while the remaining DNA was eluted by gel digestion (Gelase, Epicentre Biotechnologies, Madison, WI).

For *A. apis*, chalkbrood mummies were collected from a naturally infected honey bee colony at Weslaco, Texas. A single black mummy was pulverized in 1 ml 0.001% Trition X-100, and 50 µl of this suspension were plated on a YGPS plate (Bailey, 1981) containing ampicillin (100 µg/ml) and streptomycin (12 µg/ml). The plate was incubated for 9 days at 35 °C under 6% CO_2_, during which time fruiting bodies formed. Two millilitres of Triton X-100 were added to the plate, and the plate was rocked at 25 r.p.m. for 10 min at room temperature. One millilitre of the liquid was then mixed with 0.1 ml of 0.5 mm glass beads, and shaken at 300 r.p.m. for 1.5 h. Dilutions of this spore suspension were plated on YGPS ampicillin streptomycin plates in order to obtain single spore isolates of both mating types. After 3 days incubation, fungal colonies were picked and replated individually on fresh plates. Purified fungal colonies were tested to determine the mating type (Christensen & Gilliam, 1983).

One purified strain of each mating type was used for isolation of DNA for sequencing. One hundred-millilitre batches of liquid YGPS ampicillin streptomycin media were inoculated from purified mycelia growing on plates. Cultures were incubated for 6–9 days at 35 °C with shaking at 200 r.p.m. under normal atmosphere. Mycelial masses were removed from the cultures, and after squeezing out most of the liquid, were separated into aliquots, weighed, and stored at −80 °C until further processing. DNA was isolated from the mycelia by the method of Borges *et al*. (http://www.fgsc.net/fgn37/borges.html). The two *A. apis* isolates used in the genome sequencing project, designated 0.5–1A and A10, have been deposited in the USDA-ARSEF collection (Murray *et al*., 2005), with accession numbers 7405 and 7406, respectively.

### Genome size estimation

*Ascosphaera apis* genome size was estimated using the flow cytometry method (Bennett *et al*., 2003) and staining nuclei with propidium iodide. Briefly, spores and hyphae of *A. apis* were collected from plates in microcentrifuge tubes in 0.5 ml 0.2% Triton-X in phosphate-buffered saline (PBS). Nuclei were released by crushing spores and hyphae with a micropestle, stained with PI for at least 20 min at 4 °C. The suspension was filtered through a nylon membrane with a pore size of 15 µm to remove debris. Propidium iodide stained nuclei were analysed using a flow cytometer.
